# Staged compressive dressing method for primary giant omphalocele repair: a low-cost and successful strategy

**DOI:** 10.1007/s00383-026-06494-4

**Published:** 2026-06-15

**Authors:** Ali Ekber Hakalmaz, Rahşan Özcan, Ayşe Karagöz, Türkan Rahimli, Şenol Emre, Pınar Kendigelen, Gonca Topuzlu Tekant

**Affiliations:** Department of Pediatric Surgery, Cerrahpasa Faculty of Medicine, Istanbul University-Cerrahpasa, Cerrahpasa, Fatih, 34096 Istanbul, Turkey

**Keywords:** Giant omphalocele, Staged reduction, Compressive dressing, Primary repair, Abdominal wall defect, Low-cost treatment

## Abstract

**Purpose:**

In giant omphalocele cases, small abdominal cavity leads to high intraabdominal pressure during closure, making repair challenging. This study aimed to evaluate our staged compressive dressing technique and its outcomes.

**Methods:**

Eighteen giant omphalocele cases treated with staged compressive dressing/primary closure between 2014 and 2024 were retrospectively reviewed. All cases received compressive dressing every other day with wet sterile gauze and transparent adhesive surgical drapes from the first postnatal day until surgery to reduce intra-sac organs. Primary closure was performed after organ reduction.

**Results:**

Eighteen cases (Female/Male: 10/8) were included. Mean gestational age was 36.8 ± 1.7 weeks, birth weight 2988 ± 603 g. Major cardiac anomaly was present in 16.7% and liver herniation in 83.3%. Mean defect diameter was 6.0 ± 1.2 cm. Mean time to surgery was 6.4 ± 5.1 days. Primary repair was successfully performed in all cases (100%). Operation was delayed in patients with cardiac anomaly (13.0 ± 9.2 vs. 5.3 ± 3.4 days, *p* = 0.012). Seven patients (38.9%) had prolonged hospitalization (> 20 days). One patient with cardiac anomaly died postoperatively. Mean hospital stay was 22.2 ± 11.0 days .

**Conclusion:**

In this single-center retrospective study, the staged compressive dressing technique appeared to be a feasible, simple, and potentially cost-effective method for giant omphalocele management. While we achieved primary closure in all cases without prosthetic materials, these findings require validation through larger multicenter studies and comparative trials before definitive conclusions can be drawn about superiority over alternative methods.

## Introduction

Omphalocele is a congenital anterior abdominal wall defect characterized by herniation of intraabdominal organs (bowel, liver, stomach, etc.) through an opening in the umbilical ring within a sac composed of peritoneum, Wharton’s jelly, and amnion. Its incidence is estimated at approximately 1 in 4000 live births [1]. Omphalocele management varies depending on defect size, content of herniated organs, and associated anomalies [2]. Particularly “giant omphalocele” (GO) cases, where the defect diameter is large or a significant portion of the liver is within the sac, pose a major challenge for pediatric surgeons due to significant viscero-abdominal disproportion [3, 4].

The primary goal in GO treatment is to achieve organ reduction into the abdomen while preventing excessive elevation of intraabdominal pressure (IAP) during abdominal wall closure. High IAP can lead to abdominal compartment syndrome, hemodynamic deterioration, and respiratory failure [4, 5]. Traditional treatment methods include staged surgical closure using synthetic patches or silos (Schuster technique) and conservative “paint and wait” approaches using topical agents (silver sulfadiazine, povidone-iodine, etc.) [3, 6]. However, surgical silos carry risks of infection and fascial dehiscence, while conservative approaches are associated with giant ventral hernia formation, prolonged hospital stays, and secondary surgical requirements [7, 8].

Recently, interest has increased in non-surgical or minimally invasive staged reduction techniques (taping, hydrocolloid silos, etc.) to reduce the morbidity of invasive surgical interventions [9, 10]. In our clinic, we also apply the “staged compressive dressing” technique, which is a simple and low-cost strategy. This method aims to gradually and safely reduce herniated organs into the abdominal cavity through daily, controlled compressive dressings applied over the omphalocele sac starting immediately after birth. This reduction process is usually completed within 7 to 10 days and allows primary fascial closure without the risk of dangerous elevation of intraabdominal pressure.

This study aimed to evaluate the efficacy and safety of this low-cost and successful strategy by presenting the results of patients diagnosed with giant omphalocele who were followed in our clinic and underwent primary repair after staged compressive dressing technique.

## Materials and methods

This study was conducted with approval from the Clinical Research Ethics Committee. Data from patients followed and treated for omphalocele between 2014 and 2024 were retrospectively reviewed.

The inclusion criterion was application of the “staged compressive dressing” technique in the postnatal period for primary closure due to giant omphalocele. A basic condition for applying this technique was clinical assessment that the omphalocele sac neck (transition area between fascial defect edge and sac) was sufficiently wide to allow gradual reduction of herniated organs into the abdomen without trauma or excessive force. Cases where the sac neck was considered too narrow to allow reduction were not considered suitable for this specific method and were excluded from the study, even if the abdominal cavity was small.

Data from included patients were collected retrospectively. Recorded data included demographic and perinatal data (gender, gestational age, delivery method, birth weight, APGAR scores, prenatal diagnosis presence, maternal age), omphalocele characteristics (sac content, defect diameter), associated anomalies, treatment process (dressing duration, surgery timing), surgical findings and method, postoperative outcomes, and follow-up data.

### Subgroup analyses

Subgroup analyses were predefined to evaluate the impact of clinical variables on outcomes. Patients were stratified by: (a) gender, (b) presence of major cardiac anomaly, (c) APGAR scores (≤ 6 vs. > 6, based on the threshold for neonatal resuscitation need), and (d) operation timing (≤ 3 days for early, 4–7 days for intermediate, > 7 days for delayed repair).

### Cost estimation

Cost estimation was performed by calculating material costs for the entire treatment period. Each dressing change required: sterile gauze (approximately $2), transparent adhesive drapes ($5), and saline solution ($1), totaling approximately $8 per application. Since dressings were changed every other day, the total material cost for the average treatment duration of 6.4 days was approximately $24–32 (3–4 dressing changes).

#### Staged compressive dressing technique

Following postnatal stabilization, all patients received staged compressive dressing every two days from the first postnatal day until surgery day.

##### Technique mechanism

Our method differs from simple downward compression by creating a circumferential pressure gradient that gradually stretches abdominal wall muscles while utilizing gravity-assisted reduction. The wet gauze maintains sac moisture and tissue pliability, while adhesive surgical drapes secured over the gauze create uniform circumferential pressure. Assessment of reduction progress is performed during dressing changes every two days.

##### Patient management

All infants remained awake and spontaneously breathing without mechanical ventilation. For analgesia, we used paracetamol (10–15 mg/kg every 6–8 h). No routine sedation was required for dressing changes. In selected cases with irritability, midazolam 0.05 mg/kg IV was administered before dressing change.

##### Application protocol

(1) The omphalocele sac and surrounding area were gently cleaned with normal saline (2) Sterile gauze moistened with saline was placed over the sac (3) Self-adhesive surgical drapes were applied, providing controlled pressure.

##### Monitoring during reduction

Throughout the reduction period, patients were closely monitored for:

- Intraabdominal pressure: Assessed through abdominal distension, lower extremity perfusion, and urine output.
- Membrane integrity: Inspection for sac tears or rupture every dressing change.
- Cardiorespiratory stability: Continuous monitoring of vital signs, oxygen saturation, and feeding tolerance.

**Fig. 1:**
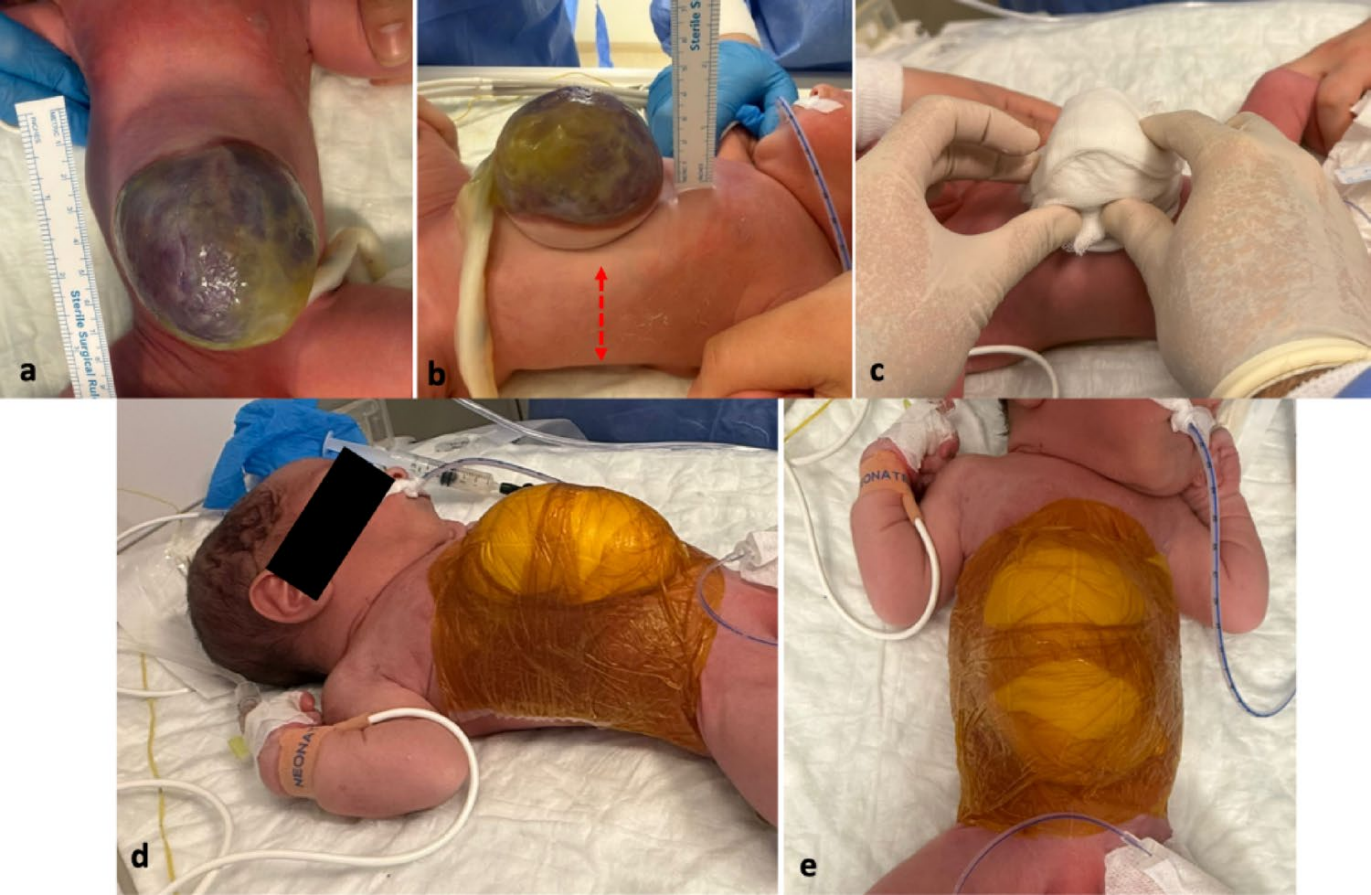
Giant omphalocele on postnatal day 1 and initial staged compressive dressing application. **a**, **b** Pre-treatment appearance showing intact sac with liver herniation. **c** Sac preparation with saline cleaning. **d**, **e** Post-dressing appearance demonstrating complete coverage with transparent adhesive drapes creating circumferential compression

**Fig. 2:**
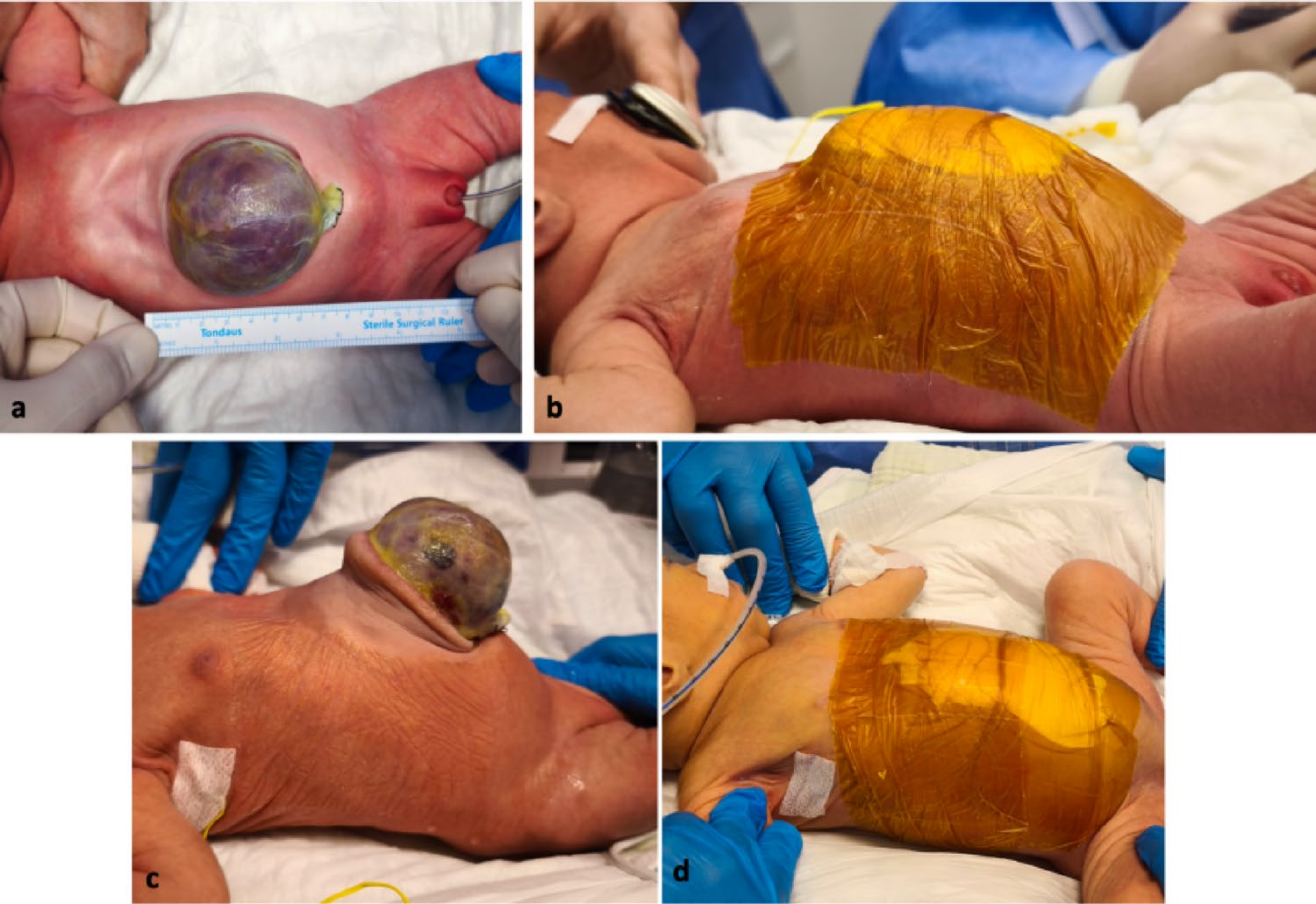
Serial progression of staged compressive dressing technique. **a**, **b** Day 2: Initial presentation with liver herniation and post-dressing application. **c** Day 4: Partial liver reduction with decreased sac prominence. **d** Day 6: Substantial organ reduction with expanded abdominal cavity capacity, ready for primary repair. Note the progressive flattening of the omphalocele and gradual abdominal wall accommodation from 2 to 6 day

Dressing was renewed every two days and pressure gradually increased based on reduction progress and patient tolerance (Figs. 1 and 2).

#### Surgical repair

**Fig. 3:**
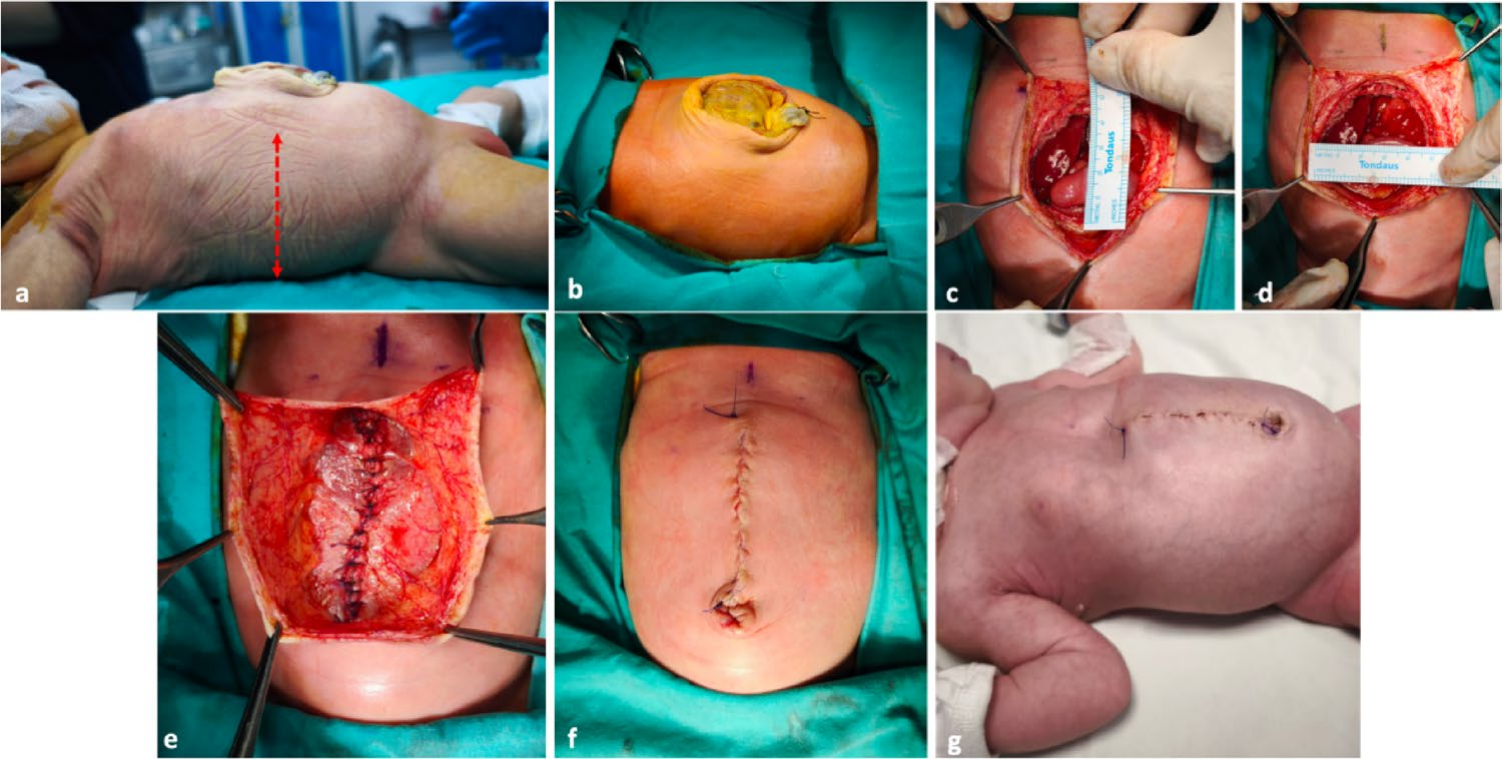
Primary repair on day 7 and postoperative outcome. **a**, **b** Preoperative view after successful compression therapy. **c**, **d** Post-excision view showing reduced liver in abdomen and fascial defect measurement. **e** Tension-free primary fascial closure. **f** Immediate postoperative cosmetic result. **g** Postoperative week 1 showing excellent healing and cosmetic outcome

Primary repair was performed when ≥ 70% of herniated contents were reduced and fascial edges could be approximated without excessive tension. Under general anesthesia, after sac excision, umbilical fascial edges were primarily repaired (Fig. 3).

### Statistical analysis

SPSS for Windows version 22.0 was used for data analysis. Descriptive statistics were presented as number (N) and percentage (%) for categorical variables; as mean ± standard deviation (SD) or median (minimum-maximum range) for continuous variables according to normality distribution. Mann-Whitney U test, Fisher’s Exact test, and Spearman correlation analysis were used for between-group comparisons. Logistic regression was applied for multivariate analysis. Statistical significance level was accepted as *p* < 0.05.

Prolonged hospitalization was defined as > 20 days based on the 75th percentile of our cohort’s length of stay distribution. Multivariable logistic regression analysis was performed to identify independent risk factors for prolonged hospitalization. Variables with *p* < 0.10 in univariate analysis were included in the model. Given our sample size limitations (*n* = 18 with 7 events), we limited the model to 3 variables to maintain statistical validity. Statistical significance level was accepted as *p* < 0.05.

## Results

Eighteen patients (10 girls, 8 boys) were included during the study period. Prenatal diagnosis was present in 16 cases (88.9%) and all deliveries were performed by cesarean section. Mean gestational age was 36.8 ± 1.7 weeks (range: 34–39 weeks), mean birth weight was 2988 ± 603 g (range: 1900–3900 g). Mean maternal age was 33.4 ± 5.4 years (23–43 years). Mean 1st minute APGAR score was 6.4 ± 1.5 (range: 3–8) and mean 5th minute APGAR score was 7.7 ± 1.2 (range: 4–9) (*n* = 17) (Table 1).

**Table 1:** Demographic, clinical and outcome data of patients with giant omphalocele treated with staged compressive dressing and primary repair (*n* = 18)

Total number of cases(*N*)	18
Gender (female/ male)	10 / 8
Prenatal diagnosis (%)	16 (88.9%)
Delivery method (cesarean, %)	18 (100%)
Mean gestational age (weeks)	36.8 ± 1.7 (34–39)
Mean birth weight (grams)	2988 ± 603 (1900–3900)
Mean APGAR (1st min)*	6.4 ± 1.5 (3–8)
Mean APGAR (5th min)*	7.7 ± 1.2 (4–9)
Major cardiac anomaly (%)	3(16.7%)
Liver herniation (%)	15 (83.3%)
Mean defect diameter (cm)^†^	6.0 ± 1.2 (4–8)
Mean time to surgery (days)	6.4 ± 5.1 (2–24)
Primary repair rate (%)	18 (100%)
Silo / patch use (%)	0 (0%)
Mortality (%)	1 (5.6%)
Postoperative incisional hernia (%)	2 (11.1%)
Mean hospital stay (days)	22.2 ± 11.0 (11–48)
Mean follow-up period (years)	3.5 (2–10 years)

*APGAR scores available for 17 patients

†Defect diameter was measured in 12 patients where complete visualization was possible

**Fig. 4:**
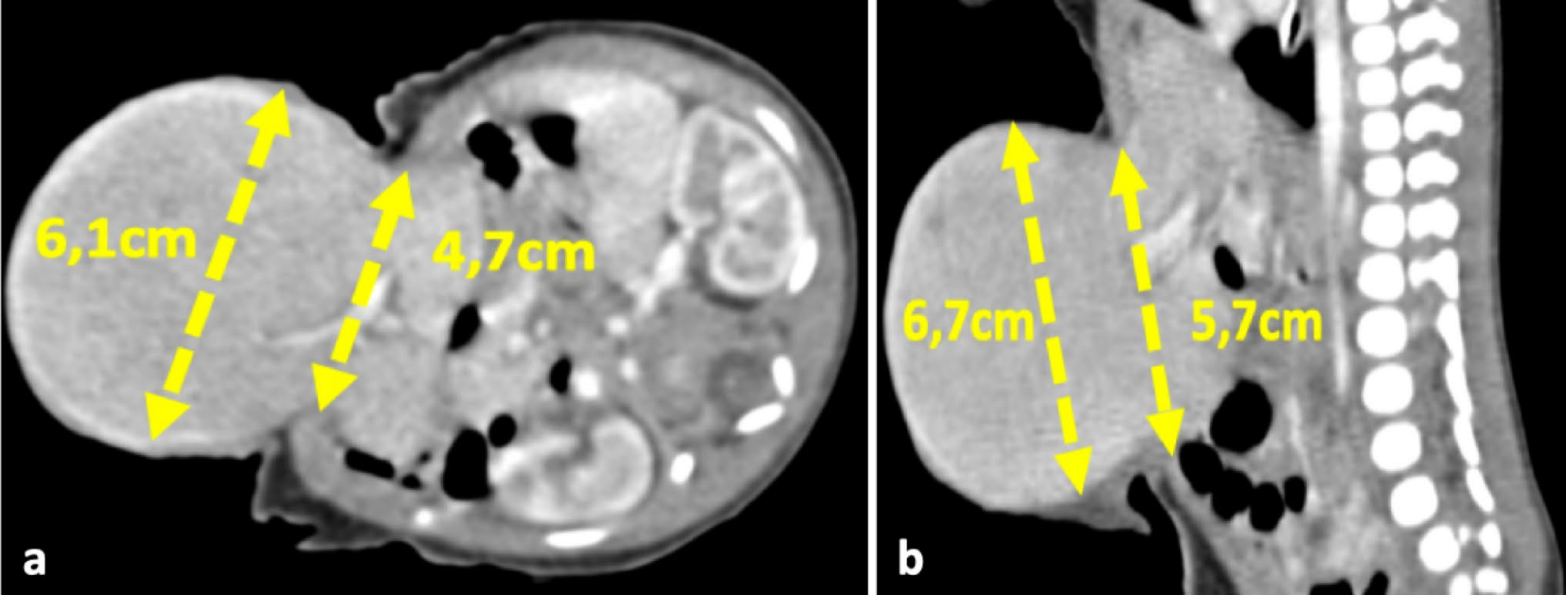
Measurement of defect diameter. **a** Case 1 showing measurement technique with ruler demonstrating 6 cm defect diameter with liver herniation visible, **b** Case 2 with 8 cm defect diameter showing extensive liver protrusion through the fascial defect

Major cardiac anomaly was detected in 16.7% (*n* = 3) of patients. Other comorbidities included one case of undescended testis, one case of mild hydronephrosis, and two cases of patent foramen ovale that closed spontaneously. Examining the contents of the omphalocele sac, liver herniation was observed in 15 patients (83.3%) and only intestinal structures in 3 patients (16.7%). In 12 patients with known defect diameter, mean umbilical fascial defect diameter was 6.0 ± 1.2 cm (range: 4–8 cm) (Fig. 4).

Mean surgery time with staged compressive dressing technique was 6.4 ± 5.1 days (range: 2–24 days). Among operation timing groups, 7 patients (38.9%) underwent early repair (≤ 3 days), 6 patients (33.3%) intermediate repair (4–7 days), and 5 patients (27.8%) delayed repair (> 7 days). Primary fascial closure was achieved in all 18 patients (100%) without fascial discharge incisions (100%). The gradual stretching from our technique provided sufficient abdominal accommodation. No silo bags or prosthetic patches were needed during treatment in any patient.

No major complications occurred during the staged reduction period. Specifically:

- No excessive intraabdominal pressure elevation requiring cessation of treatment.
- No membrane tears or sac ruptures during dressing application.
- No episodes requiring reduction abandonment due to patient instability.

No significant difference was found between female and male infants regarding operation age (6.6 ± 5.8 vs. 6.1 ± 4.3 days, *p* = 0.891), hospital stay (24.6 ± 13.2 vs. 19.1 ± 7.4 days, *p* = 0.453), and birth weight (3104 ± 647 vs. 2842 ± 541 g, *p* = 0.367).

Operation age was significantly later in patients with major cardiac anomaly (13.0 ± 9.2 vs. 5.3 ± 3.4 days, *p* = 0.012).

Seven patients (38.9%) had prolonged hospitalization (> 20 days).Hospital stay was significantly longer in patients with low APGAR scores (≤ 6) (27.6 ± 14.9 vs. 17.4 ± 4.7 days, *p* = 0.045).

When patients were divided into three groups according to operation age (≤ 3 days, 4–7 days, > 7 days), those operated late had significantly longer hospital stays (34.5 ± 17.4 vs. 16.7 ± 6.9 days, *p* = 0.033) and higher rates of major cardiac anomaly (60% vs. 0% in early repair group, *p* = 0.041) .

Independent risk factors predicting prolonged hospital stay (> 20 days) in multivariate analysis were: late operation (> 7 days) (OR:15.0, 95% CI: 1.2-187.5, *p* = 0.024), presence of major cardiac anomaly (OR:8.7, 95% CI: 1.5–50.3, *p* = 0.017), and low APGAR score (≤ 6) (OR:4.2, 95% CI: 1.1–16.8, *p* = 0.045) (Table 2).

**Table 2:** Multivariate analysis: risk factors predicting prolonged hospital stay (> 20 days) in giant omphalocele patients

Risk factor	Odds ratio (OR)	%95 confidence interval	*p* value
Late operation (> 7 days)	15.0	1.2-187.5	**0.024**
Major cardiac anomaly	8.7	1.5–50.3	**0.017**
Low APGAR (≤ 6)	4.2	1.1–16.8	**0.045**
Large defect (> 6 cm)	2.8	0.6–13.2	0.183

p< 0.05 is significant

No early or late surgical complications were detected during postoperative follow-up. Only one patient died in the entire series (5.6%, 1/18). This patient had major cardiac anomaly (ventricular septal defect and pulmonary hypertension), representing a mortality rate of 33.3% (1/3) among patients with cardiac anomalies, while no mortality was observed in those without cardiac anomaly (0/15).Two patients (11.1%) developed small (< 1 cm) incisional hernias during follow-up. Both were managed conservatively with watchful waiting, as the defects were asymptomatic and showed no progression over 12 months of follow-up.

The average total material cost for the staged compressive dressing technique was approximately $24–32 (3–4 dressing changes over 6.4 days × $8/change), compared to reported costs of $500–1000 for commercial silo bags and $2000–5000 for VAC therapy systems in literature.

Mean hospital stay was calculated as 22.2 ± 11.0 days (range: 11–48 days). Follow-up period ranged from 2 to 10 years.

## Discussion

Giant omphalocele management is a complex process due to associated anomalies and viscero-abdominal disproportion. The staged compressive dressing technique applied in our study achieved 100% primary closure success without requiring any prosthetic material or surgical silo use, even in challenging cases with small abdominal cavity and liver herniation (83.3% liver positivity). These results are consistent with high success rates and early fascial closure times reported by authors using similar minimally invasive methods such as Kogut (taping method) and Abello (hydrocolloid silo) [5, 9].

The non-surgical staged reduction strategy we applied shows significant differences from other staged methods described in literature in terms of basic principles and invasiveness. The most widely used traditional method for giant omphaloceles is the Schuster technique, which involves suturing a prosthetic material (silastic silo, etc.) to fascial edges [6, 11]. Although it provides effective visceral reduction, placement of these surgical silos requires general anesthesia and can lead to complications such as infection, silo dehiscence, or fascial edge deterioration at the suture line.

In contrast, our compressive dressing method and similar taping or hydrocolloid silo techniques in literature do not require any suturing to the fascia. Abello et al. emphasized that non-surgical adhesive silos protect the amnion and skin by distributing traction force across the entire surface rather than just the suture line, thus preserving fascial integrity for final closure [5].

Another method gaining popularity recently is Vacuum-Assisted Closure (VAC) systems, which are also used for staged reduction [12]. Although Nissen et al. reported that VAC treatment provides effective fascial approximation, infants in this series were initially intubated and required mechanical ventilator support. However, our compressive dressing technique and similar non-invasive methods (neoprene binders, elastic bandages) have the advantage of being applicable while the infant is awake and breathing spontaneously. A study using neoprene binders reported that infants required no sedation and parents could establish early contact with their babies [10]. This reduces intensive care hospitalization costs and offers a cost-effective alternative applicable even in low-resource settings compared to methods requiring expensive equipment or special consumables like VAC [4, 5].

Our technique’s material costs (approximately $25–50 for the entire treatment course with dressing changes every two days) represent a 10–100 fold reduction compared to silo or VAC alternatives, making it particularly valuable for resource-limited settings. The materials used (sterile gauze and surgical drapes) are inexpensive, and performing dressing changes every other day rather than daily further reduces material costs by approximately 50%, while still maintaining effective gradual organ reduction. Additionally, this cost advantage is amplified by avoiding intensive care interventions, mechanical ventilation, and expensive consumables associated with alternative methods.

Regarding mortality and morbidity, the single death (5.5%) in our series was related to major cardiac anomaly and pulmonary hypertension (PH). Literature emphasizes that PH incidence is high in infants with omphalocele (around 30%-50%) and is one of the independent and most important risk factors for mortality [13]. In Nolan et al.‘s study, it was demonstrated that major cardiac anomaly, low birth weight, and preterm birth were the most important factors predicting adverse outcomes in 35 giant omphalocele patients, and that patients who underwent primary closure had shorter hospital stays and the advantage of repair at an earlier age [14]. Our findings, showing delayed operative timing in patients with cardiac anomalies and the profile of the deceased patient, support the literature indicating that accompanying comorbidities rather than surgical technique determine prognosis.

### Study limitations and future perspectives

The most important limitation of our study is its single-center design and relatively small sample size (*n* = 18). Larger multi-center studies with broader patient series are needed to fully evaluate the effectiveness of this technique and prove the generalizability of our results. Particularly for rare giant omphalocele cases, more comprehensive series including different geographical regions, socioeconomic conditions, and center experiences could better demonstrate the global applicability of this low-cost technique.

Additionally, prospective randomized comparison of the staged compressive dressing technique with other treatment modalities (surgical silo, VAC treatment, conservative approach) in future studies would be valuable for determining optimal indication areas for each method. Larger-scale studies including cost-effectiveness analysis, hospital stay duration, family satisfaction, and long-term quality of life parameters would clarify this technique’s place in clinical practice.

In conclusion, the staged compressive dressing technique appears to be a method with simple applicability, low cost, and low complication rate in our experience. Although surgical silo and VAC applications may accelerate visceral reduction, the anesthetic burden and surgical trauma risk they bring should not be overlooked. The method in our study potentially minimizes these risks by providing physiological reduction of organs through gravity and external pressure without requiring invasive procedures, allowing early primary closure. However, these promising results require external validation and comparative studies before broader adoption can be recommended.

## Data Availability

No datasets were generated or analysed during the current study.
